# Evaluation of Mental Foramen with Cone Beam Computed Tomography: A Systematic Review of Literature

**DOI:** 10.1155/2021/8897275

**Published:** 2021-01-06

**Authors:** Antoinette Pelé, Pierre-Alexandre Berry, Charles Evanno, Fabienne Jordana

**Affiliations:** ^1^Oral and Maxillofacial Radiology, Dentistry Department, University Health Centre, University of Nantes, Orvault, France; ^2^Private Practice, 34 Rue Jules Verne, 44700 Orvault, France; ^3^Oral Implantology, Dentistry Department, University Health Centre, University of Nantes, Nantes, France

## Abstract

**Purpose:**

The aim of this systematic review is to assess whether the anatomy of mental foramen is precisely evaluable with cone beam computed tomography (CBCT) before implantation in humans.

**Methods:**

A systematic review was carried out to evaluate the anatomy of mental foramen (size, position, symmetry, anterior loop, and accessory mental foramen or multiple mental foramina). According to Preferred Reporting Items for Systematic reviews and Meta-Analyses (PRISMA) guidelines, an electronic search of three databases (Medline, Web of Science, and Cochrane Library) was undertaken until June 2020 and was supplemented by manual searching. Two reviewers will independently perform the processes of study inclusion, data extraction, and quality assessment. Systematic reviews, studies about children, and case reports were excluded. Only studies using CBCT to do preoperative evaluation were selected.

**Results:**

From 728 potentially eligible articles, 72 were included in the qualitative analysis and quantitative synthesis. This systematic review provided an assessment of the anatomy of the mental foramen. The mental foramen was located mostly between the two premolars (between 50.4% and 61.95%) or apically to the second premolar (from 50.3% to 57.9%). The mean diameter of the mental foramen was bigger in males than in females; the difference between them could reach 0.62 mm. The anterior loop seemed to be longer in males (between 0.87 ± 1.81 and 7.25 ± 2.02 mm) than in females (between 0.81 ± 1.18 and 6.52 ± 1.63 mm) and with the presence of teeth (from 0.91 ± 1.18 to 2.55 ± 1.28 for dentate people and from 0.25 ± 0.61 to 2.40 ± 0.88 mm for edentate population). The anterior loop and the accessory mental foramina were detected more frequently with CBCT than panoramic X-ray: only between 0.0 and 48.6% AMFs detected with CBCT were also seen with panoramic images. *Clinical Significance.* The mental foramen (MF) is an important landmark for local anesthesia and surgical and implantology procedures. Its location, morphology, and anatomical variations need to be considered to avoid mental nerve injury. The aim of this review is to evaluate the mental foramen using CBCT through a systematic literature review to improve knowledge of this complex area for the clinician.

## 1. Introduction

The mental foramen (MF) is a strategically important landmark during implantology procedures. Its location, morphology, and anatomical variations need to be considered before surgery to avoid mental nerve injury [1].

Mental foramen is located on the mandible, a median and symmetric bone, which constitutes the low part of the face and the chin skeleton. The inferior alveolar nerve (IAN) progresses into the mandibular canal in the mandibular body on each side of the mandible. This canal opens posteriorly by the mandibular foramen and anteriorly by the mental foramen. Sometimes, small foramina in the surrounding area of the mental foramen are identified as accessory mental foramina (AMFs). This way, anteriorly, the IAN spreads into two branches: the mental nerve which goes out of the mandible through the mental foramen and enables the sensibility of the chin and the inferior lip while the incisive nerve stays into the mandible and enables the innervation of homolateral incisors and canine [2].

**Figure 1:**
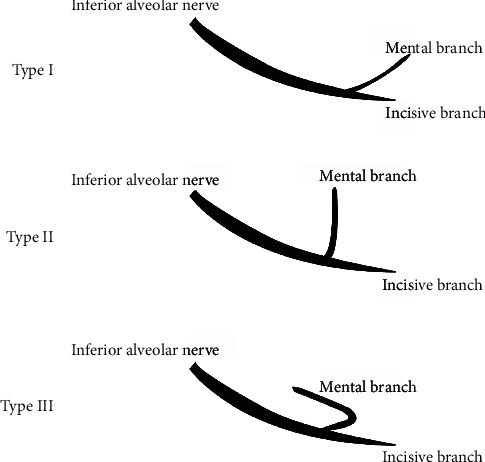
Solar et al. [3] classification of mental nerve paths. Type 1: no loop was found, and the anatomy was Y-shaped with the incisive branch usually as wide as the main branch. The mental branch leaves the inferior alveolar nerve posterior to the opening of the mental foramen [4]. Type 2: no loop was found, and the anatomy was T-shaped with the incisive branch usually as wide as the main branch. The mental branch leaves the inferior alveolar nerve perpendicular to the opening of the mental foramen [4]. Type 3: an anterior loop was found, and the anatomy was Y-shaped with the incisive branch usually as narrow as the main branch. The mental nerve branched from the inferior alveolar nerve anterior to the mental foramen [4].

The mental nerve has different ways to reach the mental foramen. Solar et al. present a classification of intraosseous part of the mental nerve with three different types (Figure 1) [3].

The accessory mental foramina may present a continuity with the mandibular canal called “accessory branch of the mandibular canal” [5].

The area of the mental foramen shows complexity, with some anatomical particularities. The nerve can be hurt in different cases like during endodontic procedures and overfilling, teeth extractions, or surgical procedure like implantology; this could create temporary sensitive consequences for the patient similar to labiomental paresthesia [5, 6]. This is why a dentist has to take all the precautions to protect the mental nerve during surgeries.

CBCT, specifically developed for imaging of the teeth and jaws [7, 8], is a low-dose scanning system [9]. CBCT use backprojection reconstructed tomography to acquire data of the whole volume of interest through a rotation of the pyramidal or conical X-ray beam and reciprocal image detector. These data are then used to generate individual slice images or 3D reconstructions [7, 9–11]. The CBCT offers a three-dimensional reconstitution of buccal cavity and different slices (axial, transversal, and sagittal) of the studied area without superposition [12].

The aim of this review is to evaluate the mental foramen using CBCT through a systematic literature review to improve knowledge of this complex area for the clinician.

## 2. Materials and Methods

The Preferred Reporting Items for Systematic Reviews and Meta-Analyses (PRISMA) system was adopted for this systematic review.

### 2.1. Study Protocol and Criteria

The protocol was designed to answer the following question: “do the CBCT studies evaluate the anatomic variations or individual parameters (sex, age, edentulism, and ethnic group) on the anatomy of mental foramen?”

It included studies reporting CBCT examination of the mental foramen and the surrounding area on patients before surgery or for routine examination or on cadavers. Some studies have also obtained results with panoramic radiography.

Inclusion criteria were as follows: only studies published in English in an international peer-reviewed journal were included. The observational studies had to describe the anatomy of mental foramen and the surrounding area using images obtained by CBCT.

Exclusion criteria were as follows: systematic reviews, studies about children, case reports, and studies using other types of computed tomography were excluded.

The following Population, Exposure to risk factor, Comparison, and Outcome (PECO) definitions were considered for systemic search:

- Population: studies had to include systemically cadavers or patients with CBCT examination
- Exposure: no risk factor
- Comparison: the specific comparisons investigated were anatomic variations or individual parameters (sex, age, edentulism, and ethnic group) on the evaluation of the anatomy of mental foramen
- Outcome measures: characteristics of mental foramen (size, position, symmetry, accessory mental foramen, or multiple mental foramina)

### 2.2. Search Strategy

A literature search was performed in MEDLINE via the PubMed database of the US National Library of Medicine, Web of Science, and Cochrane Library databases as well as a hand search of other literature to identify articles of potential relevance. The search included articles accepted for publication from 2008 up to June 2020. Previously published review articles on similar topics were also analyzed to assess potentially relevant publications.

The following keywords were used for this purpose:

  Mental foramen OR mental foramina
  AND CBCT OR Cone Beam Computed Tomography

### 2.3. Quality Assessment

Quality assurance was developed by independent screening by two reviewers (AP and PAB) according to Khan et al. [13]. When disagreement arose in the selection and eligibility, it was resolved by discussion between the two reviewers.

## 3. Results

**Figure 2:**
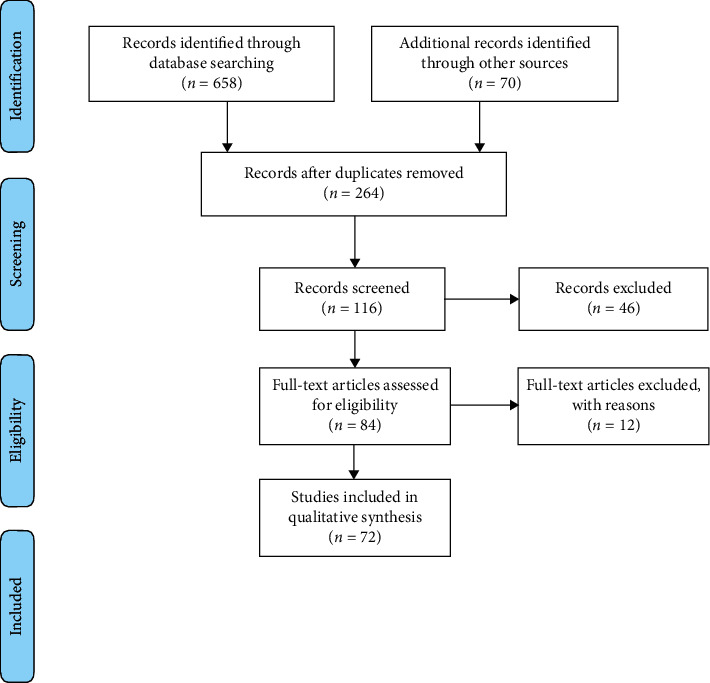
Preferred Reporting Items for Systematic Reviews and Meta-Analyses (PRISMA) flow diagram demonstrating the results of the systematic literature search.

The initial search of the literature up to June 2020 yielded 728 potentially suitable papers. Two independent reviewers (AP and PAB) analyzed titles and abstracts during the first stage of screening. Irrelevant articles and duplicates were discarded. Additional manual searching of reference lists in the papers selected as well as in a number of review articles was performed to source further relevant publications. Eighty-four full-text articles were assessed for eligibility. The full texts of the articles were read to determine whether the studies fulfilled the predetermined inclusion criteria. Seventy-two studies fulfilled the inclusion criteria and twelve were excluded (Figure 2). Following the exclusion of reviews, children studies, case reports, and studies using other types of computed tomography, 72 publications remained fully eligible for this review.

The *κ* value for interviewer agreement for study inclusion was 0.95 for titles and abstracts and 1.00 for full-text articles, indicating strong agreement.

Data was collated [4, 5, 12, 14–82] into tables (Tables 1–5 and Supplementary Tables 1–3) and grouped according to the anatomical characteristics studied. Data synthesis was performed based on the evidence tables alone, and the data was further interpreted.

### 3.1. The Mental Foramen

#### 3.1.1. Localization

Mental foramina (MFs) were equidistant from the midline of the mandible [35–37]. The mean distance did not vary much from a study to another, from around 23 mm to 26 mm [35, 37]. The interforaminal distance seemed to correspond to the double of these values, about between 46.5 ± 5 mm and 51.4 ± 0.6 mm [35, 37]. Moreover, according to Parnia et al., the mental foramen was located around 5 mm away from the lingual border of the mandible, and according to von Arx et al., the shortest distance between MF and a tooth is around 5 mm [15, 36].

When looking vertically, the MF appeared to be approximately in the middle of the mandible regardless of the side with a longer distance between the MF and mandible borders in men. The mean distance between MF and inferior border of the mandible ranged from around 10 mm and 15 mm [16, 38]. This distance was longer in male. For example, according to Gungor et al., the mean distance was 13.35 ± 2.1 mm in men, whereas it was 12.0 ± 1.8 mm in women [39].

The mean distance between MF and superior border of the mandible ranged from 11.2 ± 1.99 to 14.3 mm [12, 22]. This distance was also longer in males than in females [12, 39, 40]. For example, Gungor et al. measured 14.03 ± 2.85 mm in males against 12.53 ± 2.54 mm in females [39].

### 3.2. No Significant Difference Was Found between Right and Left Sides

The MF was located mostly between the two premolars (between 50.4% and 61.95%) or apically to the second premolar (PM2) (from 50.3% to 57.9%) [15, 16, 18–22, 25, 26]. Then, it was situated between the PM2 and the first molar (M1) (between 16.7% and 19.4%) or apically to the M1 (from 6.7% to 10.7%) and exceptionally before the PM1 or between M1 and second molar [12, 16, 19, 20, 22, 24]. Sex and side did not seem to have influence on the MF location in relation to the teeth. According to Chen et al., the presence of an anterior loop of the inferior alveolar nerve (AL) could have an influence on the position of MF to the teeth [16]. Indeed, at the majority position, without AL, the MF was found at 51.7% between PM1 and PM2, whereas when there was an AL, it was found at 73.3% apically to the PM2. On the contrary, in Krishnan et al.'s study [24], no significant difference was noticed (Table 1).

#### 3.2.1. Shape

The majority of the shapes of MF in the general population were round (between 61.57% and 72.66%) or oval (between 60.7% and 73.1%) [18, 24, 41–44], and less frequently, it could be irregular (11.14%) [25]. None of the individual parameters studied seemed to have influence of the MF shape [18, 24, 41–44].

#### 3.2.2. Diameter

The mean diameter of mental foramen ranged from 2.08 ± 0.53 mm to 4.44 ± 1.13 mm. Nevertheless, it was around 3 mm most of the time [21, 43]. The mean diameter was superior in males than in females in all the articles studied; the difference between them could reach 0.62 mm [12, 16, 25, 38–40, 45–47]. Moreover, when there was an accessory mental foramen (AMF) ipsilateral to the MF, its diameter was smaller (Supplementary Table 2) [18, 40, 48–50]. The diameter of AMF was smaller than mental foramen diameter, around 1.5 mm on average. It ranged from 0.87 ± 0.24 mm to 1.9 ± 0.6 mm [24, 40].

#### 3.2.3. Area

The mean area of MF ranged from 9.41 ± 4.58 mm^2^ to 13.1 ± 7.00 mm^2^. MF area was larger than AMF area [51, 52]. According to Muinelo-Lorenzo et al. and Iwanaga et al., the MF ipsilateral to an AMF had a smaller area (9.0 ± 5,2 and 12.9 ± 8.0 mm^2^) than the area measured in general (10.62 ± 5.00 and 13.1 ± 7.0 mm^2^) [18, 51]. On the contrary, MF was bigger on the contralateral side of an AMF or when a patient had no AMF than in general (11.36 ± 4.5 and 14.3 ± 7.2 mm^2^) [18, 51].

#### 3.2.4. Opening Angle

The mean opening angle of MF ranged from 45.65 ± 10.8 and 55.84 ± 12.16 degrees [43, 45]. According to Çağlayan et al., there was a difference between sides (about 53.58 ± 11.39° on right sides and 58.09 ± 12.93° on left sides) [53]. Moreover, in the same study, the angle varied much more: 51.14 ± 8.90° and 54.66 ± 12.48° on right sides and 58.15 ± 13.40° and 58.07 ± 12.73° on left ones, respectively, in men and in women [53].

### 3.3. The Accessory Mental Foramen

#### 3.3.1. Prevalence and Repartition

The prevalence of AMFs ranged from 2% to 26% among populations studied [26, 54]. For the sides, the maximum prevalence was 23.79%, which was quite the same than in the populations [26, 54, 55], more common in men [17, 21, 26, 29, 50, 51, 54, 56–59]. The AMF was seen more often on the right side of a patient; indeed, out of 10 studies, only three found more left sides with AMF than right ones [50, 51, 58]. Two studies found the same number of AMF on the right and left sides [26, 29, 54].

The AMF was more often found unilaterally in the studies, with a maximum of only 13 patients with AMFs on both sides in Aytugar et al.'s study [50]. Most of the time, the authors detected one to two AMFs per patient [5, 17, 24, 29, 40, 49].

#### 3.3.2. Diameter

The mean diameter of AMF ranged from 0.87 ± 0.24 mm to 1.9 ± 0.6 mm, but it was around 1.5 mm most of the time [24, 40]. So, the AMF was smaller than the MF, about a half of it. For example, according to Gümüsok et al. [47], the mean horizontal and vertical diameters of MFs were, respectively, 2.80 ± 0.99 mm and 3.11 ± 0.89, whereas there were 1.27 ± 0.40 and 1.50 ± 0.63 mm for AMFs. No difference was found between the sex of the patient or sides on the AMF mean diameter (Supplementary Table 2).

#### 3.3.3. Area

The mean area of AMF ranged from 1.20 ± 1.10 mm^2^ to 1.8 ± 1.4 mm^2^ [51, 52].

#### 3.3.4. Localization

The AMF position the most seen was posteroinferior to the MF. Indeed, most of AMFs were found at this position in 8 studies out of 17 [18, 30, 40, 47, 50, 54, 57, 60]. However, it was not the first position in each study; for example, some authors did not find any AMF at this position [29]. The AMF was located from around 2.54 ± 1.09 mm to 8.7 ± 4.3 mm away from the MF, and AMF was situated in a radius of 1 cm around the MF [49, 51]. According to Sisman et al., eight AMF showed continuity with the mandibular canal out of 14 [54]. Among them, two were connected with the AL and six were directly connected to the mandibular canal posteriorly to the MF.

### 3.4. The Mandibular Canal

Two articles mentioned three paths of the MC to the mental foramen [22, 27]. First, there were “straight” type and “perpendicular” or “vertical” type and then less frequently the AL with 15.2% and 21.96% (Table 2) [22, 27].

According Solar et al.'s classification, the paths of the MC were in frequency order: type III (AL + Y), type II (T), and type I (Y) [3, 4, 34] (Table 3). Moreover, they had another type called “others,” including other forms of AL seen at 26.8% [34].

### 3.5. The Anterior Loop of the Inferior Alveolar Nerve

#### 3.5.1. Prevalence and Repartition

Within the different studies, the prevalence of the AL ranged from 10.4% to 94% and from 10% to 86% among sides observed [61–64]. The sex of the patient did not seem to have a significant influence on it. However, ALs were situated mostly on the right side of patient in six studies out of eight (that looked at this parameter) [4, 27, 32, 36, 65, 66]. The AL could be found unilaterally or bilaterally with no predominance of one or another.

#### 3.5.2. Length

The mean AL length ranged from 0.89 ± 1.17 mm to 7.61 ± 1.81 mm [16, 38]. Among the extreme values, except for a minimal length of 0.0 mm meaning the absence of the AL, the smallest AL measured within the studies had a length of 0.15 mm, whereas the longest AL was equal to about 9.37 mm according to Yoon and al. [14, 52, 67]. The AL seemed to be longer in males (between 0.87 ± 1.81 and 7.25 ± 2.02 mm) than in female (between 0.81 ± 1.18 and 6.52 ± 1.63 mm); indeed, there were only two studies out of thirteen with a mean AL length bigger in females [16, 27]. The presence of teeth could be in favor of longer average length (from 0.91 ± 1.18 to 2.55 ± 1.28 for dentate people and from 0.25 ± 0.61 to 2.40 ± 0.88 mm with edentulous) [14, 67, 68]. Finally, the lengths measured on the left sides tended to be smaller except for Uchida et al. and Koivisto et al., who measured, respectively, 2.1 ± 1.9 mm and 3.5 mm on average against 1.7 ± 1.3 mm and 2.5 mm on the right sides (Supplementary Table 3) [62, 68].

#### 3.5.3. Angle

Only one study measured the angle of the AL. Chen et al. found a mean angle of 19.13 ± 26.89° in the population [16]. This angle appeared to be bigger on the right sides than on left ones and in females (25.21 ± 28.89°) than in males (13.06 ± 23.43°).

### 3.6. Comparison between CBCT and PAN

Mean distances between MF and midline of the mandible and interforaminal mean distance measured with CBCT were greater than those measured with PAN. For example, Madrigal et al. [35] measured 46.5 ± 5 mm between the two MFs with CBCT, while they measured 41.9 ± 7.1 mm with PAN. It was the opposite for the AL mean length (Table 4) [31, 32, 35].

Moreover, AL and AMFs were detected more frequently with CBCT. For example, according to Vujanovik-Eskenazi et al., the AL was seen in 40 patients with CBCT against 30 with PAN [31]. Only 15.79% of AMFs detected with CBCT were also seen with PAN according to Neves et al. [30] (Table 5), and this could be explained by the small diameter of AMF (often inferior to 1 mm) and was spot with difficulty on panoramic radiograph [5].

## 4. Discussion

The mental foramen is a strategically important landmark during surgical procedures [1]. Its location, number of foramina, and possibility of anterior loop of the mental nerve or AMF being present need to be considered before surgery to avoid mental nerve injury. This article systematically reviews the literature with respect to the mental foramen to reduce unintentional damage to the mental nerve.

The damage to mental foramen or inferior alveolar nerve during oral surgery or implant placement is a serious complication [83]. The incidence of mental neurosensory disturbances resulting from orthodontic, periodontal, and surgical procedures cannot be determined [84]. Juodzbalys et al. reported the complication incidence, which varies from 0% to 40%, of implant related inferior alveolar nerve (IAN) injuries [83]. The IAN is the most commonly injured nerve (64.4%), followed by the lingual nerve (28.8%) [85]. The damages could be range from mild paresthesia or dysesthesia to complete anesthesia and/or pain [86]. Many functions (speech, eating, kissing, drinking, etc.) will be affected [87]. The damage can result from the traumatic local anaesthetic injections or during the dental implant site osteotomy or placement or direct injury with scalpel with extreme alveolar process resorption [83].

CBCT allows collecting extensive informations about the MF anatomy, its environment and anatomical variations that can exist when some clinical parameters vary as sex, side or dental status.

When comparing two- and three-dimensional imaging, some differences were noticed. The average distances between MF and midline and between the mental foramina measured with cone beam were greater than those measured with PAN [35]. It was the contrary for AL lengths [31, 32]. CBCT overcomes certain limitations of two-dimensional imaging, such as distortion, magnification, and superimposition [88]. CBCT-reformatted panoramic images have been shown to be superior to PAN identifying the mandibular canal, because these images are free of magnification and superimposition [89]. So, according to Aminoshariae et al., CBCT is better in detecting anatomical particularities of each patient [90]. The authors supported the use of CBCT to evaluate the patient anatomy to avoid nerve injury before surgery [91].

To avoid nerve injury during surgery in the foraminal area, guidelines were developed based on the literature with respect to verifying the position of the mental foramen and validating the presence of an anterior loop of the mental nerve according to Greenstein and Tarnow [1]. The practitioner must be aware of the mental area anatomy and morphology before undertaking any treatment [84]. According to Juodzbalys et al. [83], the best way to prevent these damages is to have a clear three-dimensional vision of the jaw: proper presurgery planning, timely diagnosis, and treatment are the key to avoiding nerve sensory disturbances management. These guidelines included leaving a 2 mm safety zone between an implant and the coronal aspect of the nerve, three-dimensional radiographic evaluation, or surgical corroboration of the mental foramen's position [1].

Clinicians must also know the opening angle of the MF when operating on this area. For example, the diameter and length of an implant could be modified according to this angle. A smaller or thinner implant could be necessary when the opening angle of the MF increases since it means that the MF opens at a higher level than the mandibular canal one.

## 5. Conclusion

In dentistry, the acknowledgment of the anatomy of the oral cavity is necessary to treat patients correctly, especially for local anesthesia, in surgery or implantology consequently risking damage to critical structures, such as nerves and blood vessels. However, each person presents particularities, the anatomic variations, increasing the risks during our intervention. So, the clinician has to detect them during the preoperative phase to organize the patient treatment.

In the systematic review, the advantages of the CBCT technique were studied to analyze a surgical zone at anatomical risk within the mandible, that is, the mental foramen. The mental foramen was located mostly between the two premolars (between 50.4% and 61.95%) or apically to the second premolar (from 50.3% to 57.9%). The mean diameter of the mental foramen was bigger in males than in females, with a difference reaching 0.62 mm. The mean AL length ranged from 0.89 ± 1.17 mm to 7.61 ± 1.81 mm. The prevalence of the AL ranged from 10.4% to 94%, and from 10% to 86% among sides observed. The AMF, most seen in posteroinferior location to the MF, was situated from around 2.54 ± 1.09 mm to 8.7 ± 4.3 mm away from the MF.

## Figures and Tables

**Table 1:** Location of the mental foramen (MF) in relation to adjacent teeth *n* MF (%).

Articles	Anterior to PM1	In line with PM1	Between PM1 and PM2	In line with PM2	Between PM2 and M1	In line with M1 (often to the mesial root)	Between M1 and M2	Total
[14]	0 (0.0)	18 (4.8)	231 (59.8)	120 (30.4)	16 (4.2)	1 (0.3)	0 (0.0)†	386 (100)
[15]	0 (0.0)	7 (4.2)	94 (56)	60 (35.7)	7 (4.2)	0 (0.0)	0 (0.0)	168 (100)
[16]	(0.0^†^)	(3.33)	(51.67)	(40.83)	(4.17)	(0.0^†^)	(0.0^†^)	(100)
	—	(6.66^†^)^‡^	(20)^‡^	(73.33)^‡^	0^‡^	—	—	(100)
[17]	57 (53.8)			1 (1)	48 (45.3)			106 (100)
[18]	0 (0.0)†	32 (5)	161 (25.3)	369 (57.9)	58 (9.1)	17 (2.7)	0 (0.0)†	637 (100)
[19]	0 (0.0)	1 (2.6)	9 (23.1)	22 (56.4)	7 (17.9)	0 (0.0)	0 (0.0)†	39 (100)
[20]	0 (0.0)†	0† (0.0†)	103 (33.0)	157 (50.3)	52 (16.7)	0 (0.0)†	0 (0.0)†	312 (100)
[21]	(0.0)†	(5.0)	(25.3)	(57.9)	(0.0)†	(2.7)	(9.1)	(100)
[22]	0 (0.0)†	62 (10.3)	179 (29.6)	319 (52.8)	36 (6)	8 (1.3)	0 (0.0)†	604 (100)
[23]	0 (0.0)†	(7.03)	(73.39†)	(17.74)	(1.83†)	(0.0†)	(0.0 †)	(100)
[12]	1 (0.6)	18 (10.7)	75 (44.4)	69 (40.8)	5 (3.0)	1 (0.6)	0 (0.0)†	169 (100)
[24]	0.0	(1.9)	(43.5)	(34.3)	(19.4)	(0.9)	(0.0†)	(100)
	—	—	(41.2)^‡^	(37.3)^‡^	—	—	—	(100)
[25]	0 (0.0)	5 (3.6)	70 (50.4)	57 (41.0)	7 (5.0)			139 (100)
[26]	2 (0.25)†	66 (8.26)†	495 (61.95)†	219 (27.41)†	11 (1.38)†	6 (0.75)†	—	799† (100)

PM1: first premolar, PM2: second premolar, M1: first molar, and M2: second molar. ^†^Values calculated based on data from publications.^‡^In the presence of an anterior loop of the inferior alveolar nerve.

**Table 2:** Path of mandibular canal (MC) to the mental foramen *n* MC (%).

Articles	Straight	Perpendicular/vertical	Anterior loop
[27]	329 (33.30)†	442 (44.74)†	217 (21.96)†
[22]	279 (46.2)	233 (38.6)	92 (15.2)

^†^Values calculated based on data from publications.

**Table 3:** Distribution of mental nerve paths *n* (%) according Solar et al. [3] classification.

Articles	Type I (Y)	Type II (T)	Type III (AL + Y)	Others (AL)	Number of sites
[4]	48 (8.6)	178 (31.9)	332 (59.5)	—	558 (100)
[34]	15 (10.6)	39 (27.5)	50 (35.2)	38 (26.8)	142 (100)

AL: anterior loop.

**Table 4:** Comparison between cone beam computed tomography (CBCT) and panoramic radiograph (PAN) in measuring distances (mm).

Articles	Mean distance MF–midline of the mandible				Mean interforaminal distance		Mean length of the AL		Mean distance AL–inferior border of the mandible	
	Right side		Left side							
	CBCT	PAN	CBCT	PAN	CBCT	PAN	CBCT	PAN	CBCT	PAN
[35]	23 ± 2.8	21.3 ± 3.5	23 ± 2.4	21 ± 3.9	46.5 ± 5	41.9 ± 7.1	—	—	—	—
[31]	—	—	—	—	—	—	1.59 ± 0.93	2.82 ± 0.91	11.43 ± 1.81	11.64 ± 1.81
[32]	—	—	—	—	—	—	2.79 ± 0.82^†^	3.03 ± 0.92^†^	—	—

MF: mental foramen. AL: anterior loop.^†^Values calculated based on data from publications.

**Table 5:** Comparison between cone beam computed tomography (CBCT) and panoramic radiograph (PAN) for the visualization of anatomical structures.

Articles	Detection of accessory mental foramina (AMFs) *n* AMFs (%)		Visualization of the anterior loop (AL) *n* AL (%)	
	CBCT	PAN	CBCT	PAN
[28]	37 (100.00)	18 (48.60)	—	—
[29]	4 (100.00^†^)	0 (0^†^)	—	—
[30]	19 (100.00^†^)	3 (15.79^†^)	—	—
[31]	—	—	40 (48.80)/patient	30 (36.60)/patient
[18]	(100.00)	(45.83)	—	—
[32]	—	—	60^1^ (30.30^†^)/side	41^1^ (20.10^†^)/side
[33]	—	—	67 (37.20)/side	102 (56.75)/side

^†^Values calculated based on data from publications.

## Data Availability

Data are included within the article.

## Supplementary Materials

Supplementary Materials Supplementary Material 1: design of selected publications. Supplementary Material 2: mean diameter of mental foramen and accessory mental foramen (mm). Supplementary Material 3: mean length of the anterior loop (mm).
