# Supplementary material for: Evaluation of Mental Foramen with Cone Beam Computed Tomography: A Systematic Review of Literature
Source: Radiol Res Pract. 2021 Jan 6;2021:8897275. doi: 10.1155/2021/8897275 (PMC7806401; doi:10.1155/2021/8897275)
Supplement: Supplementary Materials — Supplementary Material 1: design of selected publications. Supplementary Material 2: mean diameter of mental foramen and accessory mental foramen (mm). Supplementary Material 3: mean length of the anterior loop (mm). [file 8897275.f1.zip › Supplementary material 1 (1).docx]

**Supplementary Material 1.** Design of selected publications

| Author | | Design of study | Number of patients | Number of sides | | Mean age (years) | Male/female ratio | | Population | Nationality n (%) or ethny | Dental status n (%) | | Imaging techniques |
| --- | --- | --- | --- | --- | --- | --- | --- | --- | --- | --- | --- | --- | --- |
|  |  |  |  | male | female |  | male | female |  |  | Right | Left |  |
| Madrigal (2008) [35] | | In vivo | 50 | - | | - | 14 | 36 | patients | - | - | | PAN  CBCT |
| Katakami (2008) [5] | | In vivo | 150 | - | | - | - | - | patients | - | - | | CBCT |
| Naitoh (2009) [40] | | In vivo | 157 | 314 | | 51.5±14.9 | 48 | 109 | patients | - | - | | CBCT |
| Uchida (2009) [68] | | Ex vivo | 71 | 140 | | 75.4 | 38 | 29 | cadavers | Japanese | - | | CBCT |
|  |  |  |  | 75 | 58 |  |  |  |  |  |  |  |  |
| Naitoh (2010) [77] | | In vivo | 28 | 56 | | 54.5±10.9 | 6 | 22 | patients | - | - | | CBCT |
| Oliveira-santos (2011) [48] | | In vivo | 285 | - | | - | 13^†^ | 15^†^ | patients | 277 Caucasians, 8 non-Caucasians | - | | CBCT |
| Naitoh (2011) [28] | | In vivo | 365 | - | | 51.7±15.1 | 130 | 235 | patients | - | - | | PAN  CBCT |
| Kajan (2012) [65] | | In vivo | 84 | 168 | | 49 | 41 | 43 | patients | Iranian | - | | CBCT |
| Parnia (2012) [36] | | In vivo | 96 | - | | 46.60 | 50^†^ | 46^†^ | patients | - | (100)^§^ | | CBCT |
| Kalender (2012) [45] | | In vivo | 193 | 386 | | 38.6 | 92 | 101 | patients | - | 92^‡^  85^§^  16 ^¶^ | | CBCT |
| Apostolakis (2012) [67] | | In vivo | 93 | 186 | | 53 | 42 | 51 | patients | - | 90^‡^  3 ^¶^ | | CBCT |
| Ritter (2012) [46] | | In vivo | 1010 | - | | 40.9  ±20.03 | 525 | 485 | patients | - | - | | CBCT |
| Sisman (2012) [54] | | In vivo | 504 | - | | 39.73±15.88 | 307 | 197 | patients | - | - | | CBCT |
| Chen (2013) [38] | a | In vivo | 100 | - | | 53.32±12.75 | 53 | 47 | patients | American | - | | CBCT |
|  | b | In vivo | 100 | - | | 53.73 ± 13.15 | 55 | 45 | patients | Taiwanese | - | | CBCT |
| Rosa (2013) [14] | | In vivo | 352 | - | | - | 129 | 197 | patients | - | - | | CBCT |
| Göregen (2013) [49] | | In vivo | 315 | 630 | | 40.9 ± 15.9 | 196 | 119 | patients | - | - | | CBCT |
| von Arx (2013) [15] | | In vivo | 142 | 168 | | 39.7 | 62 | 80 | patients | - | - | | CBCT |
|  |  |  |  | 76 | 92 |  |  |  |  |  |  |  |  |
| Imada (2012) [29] | | In vivo | 100 | 200 | | 40.9 | 34 | 66 | patients | - | - | | PAN  CBCT |
| Filo (2014) [66] | | In vivo | 694 | 1384 | | 29.89 | 341 | 353 | patients | - | 601 (86.60) ^‡^  93 (13.40) ^§^ | | CBCT |
| Çağlayan (2014) [53] | | In vivo | 192 | - | | 32.5±13.8 | 86 | 106 | patients | - | - | | CBCT |
| Neves (2013) [30] | | In vivo | 127 | 254 | | 41.9 | 55 | 72 | patients | - | - | | PAN  CBCT |
| Khojastepour (2015) [56] | | In vivo | 156 | - | | 36.99 ± 8 | 69 | 87 | patients | Iranian | - | | CBCT |
| Sheikhi (2015) [41] | | In vivo | 180 | 360 | | 48 ± 13.9 | 84 | 96 | patients | Iranian | (75) ^§^  (17.2) ^¶^  (7.8) ^‡^ | (75) ^§^  (16.7) ^¶^  (8.3) ^‡^ | CBCT |
| Demir (2015) [4] | | In vivo | 279 | 558^†^ | | 32.68 ± 10.23 | 141 | 138 | - | Turkish | - | | CBCT |
| Chen (2015) [16] | | In vivo | 60 | - | | - | - |  | patients | - | - | | CBCT |
| Vujanovic-Eskenazi (2015) [31] | | In vivo | 82 | - | | 56.56 | 26 | 56 | patients | - | - | | PAN  CBCT |
| Carruth (2015) [17] | | In vivo | 106 | - | | - | 30 | 76 | patients | - | - | | CBCT |
| Muinelo-Lorenzo (2015) [18] | | In vivo | 344 | 688 | | 47.44 ± 15.52 | 139 | 205 | patients | - | - | | PAN  CBCT |
| Lu (2015) [78] | | In vivo | 366 | 732 | | - | 183 | 183 | patients | - | - | | CBCT |
| Sheikhi (2016) [37] | | In vivo | 180 | 360^†^ | | 48.6 | 84 | 96 | patients | - | (75.0)^§^  (17.2) ^¶^  (7.8) ^‡^ | (75.0)^§^  (16.7) ^¶^  (8.3) ^‡^ | CBCT |
| Eren (2016) [61] | | In vivo | 141 | 282 | | 41.2 ± 17.74 | 62 | 79 | patients | Turkish | - | | CBCT |
| Panjnoush (2016) [79] | | In vivo | 200 | - | | 50.10 ± 13.37^†^ | 90 | 110 | patients | - | - | | CBCT |
| Iwanaga (2016) [51] | | Ex vivo | 63 | 126 | | 79.4 ± 11.9 | 31 | 32 | mandibles cadavers | Japanese | - | | CBCT |
| Koivisto (2016) [62] | | In vivo | 106 | - | | - | 34 | 72 | patients | - | - | | CBCT |
| Sahman and Sisman (2016) [27] | | In vivo | 494 | - | | 41.2 ± 15 | 254 | 240 | patients | Turks (North-West Turkey) | - | | CBCT |
| Han (2016) [19] | | In vivo | 446 | 892 | | 41 | 217 | 229 | patients | - | - | | CBCT |
| Safaee (2016) [20] | | In vivo | 312 | 312 | | 34.2 ± 9.79 | 138 | 174 | patients | - | - | | CBCT |
| Muinelo-Lorenzo (2017) [21] | | In vivo | 344 | 688 | | 47.44 ± 15.52 | 139 | 205 | patients | - | - | | CBCT |
| Zmyslowska-Polakowska (2017) [80] | | In vivo | 200 | 400 | | 54.57 ± 10.26 | 105 | 96 | patients | Polish | - | | CBCT |
| Al-Mahalawy (2017) [22] | | In vivo | 302 | 604^†^ | | 34.9 | 196 | 106 | patients | - | - | | CBCT |
| Chong (2017) [81] | | In vivo | - | - | | - | - | - | patients | - | - | | CBCT |
| Gungor (2017) [39] | | In vivo | 210 | - | | - | 107 | 103 | patients | - | - | | CBCT |
| Kumbargere Nagraj (2017) [32] | | In vivo | 99 | 198^†^ | | 44.5 | 43 | 56 | patients | (44.4) Chinese people, (36.4) Malays,  (19.2) Indians | - | | PAN  CBCT |
| Yovchev (2017) [60] | | In vivo | 1400 | - | | 46.7 | 700 | 700 | patients | Caucasians Bulgarians | - | | CBCT |
| Yang (2017) [82] | | In vivo | 412 | 824 | | - | 166 | 246 | patients | - | - | | CBCT |
| Marieiro (2017) [83] | | In vivo | 82 | - | | 63.51 ± 9.49 | 60 | 22 | patients | - | - | | CBCT |
| Velasco-Torres (2017) [23] | | In vivo | 348 | - | | 48.57 | 172 | 176 | patients | - | 183 (52.59) ^§^  98 (28.45) ^‡^  66 (18.97) ^¶^ | | CBCT |
| Moghdam (2017) [84] | | In vivo | 234 | 452 | | 50.1 ± 13.3 | 113 | 121 | patients | Iranian | - | | CBCT |
| Aoun (2017) [85] | | In vivo | 50 | 100 | | 23.46 ± 4.45 | 23 | 27 | patients | Lebanese | (100) ^‡^ | | CBCT |
| Gümüsok (2017) [47] | | In vivo | 645 | - | | 41 | 281 | 364 | patients | - |  | | CBCT |
| Kheir (2017) [86] | | In vivo | 180 | 360^†^ | | 48.6 | 84 | 96 | patients | Iranian | (75.0)^§^  (17.2) ^¶^  (7.8) ^‡^ | (75.0) ^§^  (16.7) ^¶^  (8.3) ^‡^ | CBCT |
| Shaban (2017) [34] | | In vivo | 71 | 142 | | 43.54 ± 9.72 | 36 | 35 | patients | - | - | | CBCT |
| dos Santos Oliveira (2018) [12] | | In vivo | 104 | 169 | | 49.2 | 32 | 72 | patients | - | - | | CBCT |
|  |  |  |  | 48 | 121 |  |  |  |  |  |  |  |  |
| Alam 2017) [42] | | In vivo | 395 | - | | - | 268 | 127 | patients | Arabs (321 Saudis,  42 Jordanians,  32 Egyptians) | - | | CBCT |
| Li (2018) [57] | | In vivo | 784 | 1568^†^ | | 34 | 305 | 479 | patients | Chinese | - | | CBCT |
| Krishnan (2018) [24] | | In vivo | 109 | - | | 40.5 | 52^†^ | 57^†^ | patients | 8.5% Asians,  12.3% black, people, 69.8% white people,  9.4% not available | - | | CBCT |
| Christopher (2018) [63] | | In vivo | 85 | 140 | | - | - | - | patients | - | - | | CBCT |
| Wong (2018) [64] | | In vivo | 100 | - | | - | - | - | patients | 33 Malays,  33 Indians, 34 Chinese | - | | CBCT |
| do Carmo Oliveira (2018) [87] | | In vivo | 202 | - | | - | 61 | 141 | patients | - | - | | CBCT |
| Goyushov (2018) [43] | | In vivo | 663 | 1326 | | - | 272^†^ | 391 | patients | Caucasians | - | | CBCT |
| Alsoleihat (2018) [25] | | In vivo | 139 | - | | 43.5 | 50 | 89 | patients | Jordanian | - | | CBCT |
| Vieira (2018) [88] | | In vivo | 240 | 480 | | 46.6 ± 17.1 | 77 | 163 | patients | - | - | | CBCT |
| Kastala (2019) [33] | | In vivo | 180 | 360 | | - | 45 | 45 | patients | - | - | | PAN  CBCT |
| Zmyslowska (2019) [44] | | In vivo | 201 | - | | - | 106 | 95 | patients | - | - | | CBCT |
| Aytugar (2019) [50] | | In vivo | 1005 | - | | 39.98±17.39^†^ | 503 | 502 | patients |  | (100) ^‡^ | | CBCT |
| Bosykh (2019) [26] | | In vivo | 400 | - | | 42.26 ± 13.41 | 190 | 210 | patients | Caucasian Russians | - | | CBCT |
| Lam (2019) [58] | | In vivo | 4000 | - | | - | - | - | patients | Australian | - | | CBCT |
| Xie (2019) [89] | | In vivo | 1008 | - | | 41.1±14.81 | 521 | 487 | patients | Chinese | - | | CBCT |
| Yoon (2019) [52] | | In vivo | 200 | 400 | | 55 | 85 | 115 | patients | 139 white patients,  32 Hispanics,  16 Asians,  13 African Americans | - | | CBCT |
| Wei (2019) [59] | | In vivo | 306 | 612 | | 41 | 134 | 172 | patients | Southern Chinese people | - | | CBCT |
| Valdec (2019) [90] | | In vivo | 314 | - | | - | 146 | 168 | patients | - | - | | CBCT |
| Raju (2019) [55] | | In vivo | 124 | 248 | | - | 56 | 68 | patients | - | 98^‡^  26 ^¶^ | |  |

PAN: panoramic radiograph

CBCT: cone beam computed tomography

^†^: Values calculated based on data from publications

Dental status of patients: ^‡^: dentate, ^§^: partially edentate, ^¶^: fully edentulous.
