# Supplementary material for: Evaluation of Mental Foramen with Cone Beam Computed Tomography: A Systematic Review of Literature
Source: Radiol Res Pract. 2021 Jan 6;2021:8897275. doi: 10.1155/2021/8897275 (PMC7806401; doi:10.1155/2021/8897275)
Supplement: Supplementary Materials — Supplementary Material 1: design of selected publications. Supplementary Material 2: mean diameter of mental foramen and accessory mental foramen (mm). Supplementary Material 3: mean length of the anterior loop (mm). [file 8897275.f1.zip › Supplementary material 2 (1).docx]

**Supplementary Material 2.** Mean diameter of mental foramen and accessory mental foramen (mm)

| Articles | | MF | AMF | | MF | | AMF | MF | | AMF | MF | | AMF | MF | | AMF | MF | |
| --- | --- | --- | --- | --- | --- | --- | --- | --- | --- | --- | --- | --- | --- | --- | --- | --- | --- | --- |
|  |  | general case | | | right | | | left | | | males | | | females | | | ipsilateral to the AMF | controlateral to the AMF or sides without AMF |
| [40] | | - | | 1.9 ± 0.6 ^¶^ | 3.24±0.63^†§^ | - | | 3.31±0.86^†§^ | - | | 3.54±0.73^†§^ | - | | 3.16±0.75^†§^ | - | | 3.8 ± 0.9 ^¶^ | 4.2 ± 0.5 ^¶^ |
| [48] | | - | | - | - | - | | - | - | | - | - | | - | - | | 3.83± 0.65 | 4.13 ± 0.62 |
| [45] | | 3.4^‡^  3.7^§^ | | 1.55±0.55^†‡^  1.4 ±0.35^†§^ | 3.8 ± 0.8^‡^  3.5 ± 0.8^§^ | 1.5 ± 0.5^‡^  1.4 ± 0.3^§^ | | 3.7 ± 0.8^‡^  3.4 ± 0.8^§^ | 1.6 ± 0.6^‡^  1.4 ± 0.4^§^ | | 3.95 ±0.8^†‡^  3.65 ±0.8^†§^ | 1.55 ±0.5^†‡^  1.45 ±0.4^†§^ | | 3.55±0.8 ^1‡^  3.3 ± 0.7^†§^ | 1.55 ±0.6^†‡^  1.35 ±0.5^†§^ | | - | - |
| [46] | | 3.4 ± 0.88 | | - | - | - | | - | - | | 3.6 ± 0.81 | - | | 3.3 ± 0.78 | - | | - | - |
| [38] | a | 2.26 ± 0.67 | | - | - | - | | - | - | | 2.24± 0.62 | - | | 2.14 ± 0.53 | - | | - | - |
|  | b | 2.13 ± 0.47 | |  |  |  |  |  |  |  |  |  |  |  |  |  |  |  |
| [49] | |  | | - | - | - | | - | - | | - | - | | - | - | | 4.2 ± 1.2^§^ | 4.5 ± 1.3 |
| [15] | | 3.1^‡^  2.9^§^ | | - | - | - | | - | - | | - | - | | - | - | | - | - |
| [29] | | 2.34 ± 0.4 | | - | - | - | | - | - | | - | - | | - | - | | - | - |
| [37] [41] | | 3.59 ±1.96^†^ | | - | 3.59 ± 2.74 | - | | 3.59 ± 1.17 | - | | - | - | | - | - | | - | - |
| [16] | | 2.95 ± 0.59 | | - | 2.82 ± 0.57 | - | | 3.07 ± 0.58 | - | | 3.08 ± 0.55 | - | | 2.81 ± 0.60 | - | | - | - |
| [18] | | - | | 1.80±0.66 ^¶^  1.12±0.39^#^ | - | - | | - | - | | - | - | | - | - | | 4.0 ± 1.2 ^¶^  2.6 ± 0.7^#^ | 4.7 ± 0.9 ^¶^  3.0 ± 0.7^#^ |
| [19] | | - | | 1.54±1.41^‡^  1.44±0.41^§^ | - | - | | - | - | | - | - | | - | - | | - | - |
| [21] | | 4.44 ±1.13^¶^  2.92±0.75^#^ | | - | - | - | | - | - | | - | - | | - | - | | - | - |
| [39] | | - | | - | 3.56 ±0.68^‡^  3.12 ±0.74^§^ | - | | 3.31 ±0.66^‡^  3.12 ±0.64^§^ | - | | 3.56 ±0.72^‡^  3.31 ±0.73^§^ | - | | 3.31 ±0.62^‡^  2.93 ±0.59^§^ | - | | - | - |
| [60] | | - | | 1.5±0.4 ^¶^  1.2 ±0.3^#^ | - | - | | - | - | | - | - | | - | - | | - | - |
| [47] | | 2.80 ±0.99^‡^  3.11 ±0.89^§^ | | 1.27±0.40^‡^  1.50±0.63^§^ | - | - | | - | - | | 2.89 ±0.97^‡^  3.31 ±1.01^§^ | 1.27±0.45^‡^  1.50 ±0.51^§^ | | 2.75 ±0.84^‡^  3.03 ±0.83^§^ | 1.27±0.37^‡^  1.50±0.59^§^ | | - | - |
| [12] | | 3.20 ±0.88^‡^  3.11 ±0.75^§^ | | - | - | - | | - | - | | 3.32 ±0.86^‡^  3.41 ±0.93^§^ | - | | 3.14 ±0.89^‡^  2.99 ±0.63^§^ | - | | - | - |
| [57] | | - | | 1.38±0.47^‡^  1.23±0.37^§^ | - | 1.35±0.42^‡^  1.23±0.52^§^ | | - | 1.45±0.59^‡^  1.26±0.38^§^ | | - | 1.36±0.53^†‡^  1.24±0.39^†§^ | | - | 1.38±0.40^†‡^  1.21±0.36^†§^ | | 3.10±0.55^‡^  2.91±0.47^§^ | - |
| [24] | | 3.10 ±0.86^‡^  2.84 ±0.94^§^ | | 0.87±0.24 | - | - | | - | - | | - | - | | - | - | | - | - |
| [43] | | 2.08±0.53^†§^ | | - | 2.16 ± 1.9^§^ | - | | 2.07±0.54^§^ | - | | - | - | | - | - | | - | - |
| [25] | | - | | - | - | - | | - | - | | 3.08 ±0.65^¶^ | - | | 2.46 ±0.58^¶^ | - | | - | - |
| [50] | | - | | 1.74±0.75^‡^  1.36±0.54^§^ | - | - | | - | - | | - | 1.84±0.81^‡^  1.39±0.46^§^ | | - | 1.59±0.63^‡^  1.32±0.63^§^ | | 3.57±1.15^‡^  3.21±0,86^§^ | 3.98±1.10^‡^  3.68±0.85^§^ |
| [52] | | 4.11±1.07^¶^  2.80±0.72^#^ | | 1.45±0.62^¶^  0.92±0.46^#^ | - | - | | - | - | | - | - | | - | - | | - | - |

MF: mental foramen

AMF: accessory mental foramen

^†^: values calculated based on data from publications

^‡^: horizontal

^§^: vertical

^¶^: long

^#^: short
