# Supplementary material for: Evaluation of Mental Foramen with Cone Beam Computed Tomography: A Systematic Review of Literature
Source: Radiol Res Pract. 2021 Jan 6;2021:8897275. doi: 10.1155/2021/8897275 (PMC7806401; doi:10.1155/2021/8897275)
Supplement: Supplementary Materials — Supplementary Material 1: design of selected publications. Supplementary Material 2: mean diameter of mental foramen and accessory mental foramen (mm). Supplementary Material 3: mean length of the anterior loop (mm). [file 8897275.f1.zip › Supplementary material 3.docx]

**Supplementary Material 3.** Mean length of the anterior loop (mm)

| **Articles** | | **Minimum length measured** | **Maximum**  **length measured** | **General**  **population** | **According to the sex of the patient** | | **According to the dental status of the patient** | | **According to the side** | |
| --- | --- | --- | --- | --- | --- | --- | --- | --- | --- | --- |
|  |  |  |  |  | **Males** | **Females** | **Dentate** | **Edentulous** | **Right** | **Left** |
| **[68]** | | 0.8 | 9 | 1.9± 1.7 | 2.2 ± 1.7 | 1.5 ± 1.6 | 2.1 ± 1.8 | 1.6 ± 1.4 | 1.7 ± 1.3 | 2.1 ± 1.9 |
| **[36]** | | - | - | 3.54 ±1.4 | - | - | - | - | 3.6 ± 1.0 | 3.2 ± 1.5 |
| **[67]** | | 0.0 | 5.7 | 0.89 ± 1.17 | 0.99 ±1.15 | 0.81 ± 1.18 | 0.91±1.18 | 0.25 ± 0.61 | 1.03 ± 1.17 | 0.75 ± 1.07 |
| **[46]** | | - | 1.4 ± 0.70 | 1.4 ± 0.7 | 1.6 ± 0.74 | 1.4± 0.63 | - | - | - | - |
| **[38]** | **a** | - | - | 6.22 ± 1.68 | 7.25 ± 2.02 | 6.52 ± 1.63 | - | - | - | - |
|  | **b** |  |  | 7.61 ± 1.81 |  |  |  |  |  |  |
| **[14]** | | 0.15 | 7.00 | - | - | - | 2.41±0.98 | 2.40 ± 0.88 | - | - |
| **[66]** | | 0.30 | 5.60 | 1.16 ±1.01 | 1.18 ±1.02 | 1.13 ±0.99 | 1.16 | - | 1.19 ± 0.98 | 1.13 ± 1.03 |
| **[16]** | | - | 8.41 | 1.16 ± 1.78 | 0.87 ±1.81 | 1.46 ± 1.72 | - | - | 1.22 ± 1.74 | 1.10 ± 1.83 |
| **[31]** | | - | - | 1.59 ± 0.93 | - | - | - | - | - | - |
| **[78]** | | - | 6.67 | 1.46 ± 1.25 | 1.51 ±1.24 | 1.40 ± 1.26 | - | - | 1.47 ± 1.39 | 1.44 ± 1.39 |
| **[62]** | | - | - | - | - | - | - | - | 2.5 | 3.5 |
| **[27]** | | 0.6 | 6 | - | 2.38±0.94 ^†^ | 2.66 ±0.87 ^†^ | - | - | 2.19 ± 1 | 2.08 ± 0.99 |
| **[32]** | | - | - | - | - | - | - | - | 2.87 ± 0.81 | 2.66 ± 0.84 |
| **[60]** | | 0.5 | 7.5 | 2.53 ±1.27 | 2.74 ±1.36 | 2.39 ±1.19 | 2.55±1.28 | - | - | - |
| **[23]** | | - | 7.10 | - | - | - | - | - | 2.00 ± 0.98 | 1.92 ± 0.99 |
| **[84]** | | - | - | 2.77 ± 1.56 | - | - | - | - | - | - |
| **[86]** | | 0.44 | 7.52 | - | 2.66±1.47 ^†^ | 2.39 ±1.25^†^ | - | - | 2.69 ± 1.56 | 2.36 ± 1.16 |
| **[24]** | | - | - | 3.38 ± 0.96 | - | - | - | - | - | - |
| **[63]** | | 1.75 | 3.58 | 2.79 | - | - | - | - | - | - |
| **[64]** | | 0.73 | 7.99 | 2.81 ±1.74 ^†^ | 4.1 ±1.69^†^ | 3.44 ±1.85 ^†^ | - | - | 3.85 ± 1.73 | 3.69 ± 1.75 |
| **[87]** | | - | - | 2.12 | 2.43 ±1.05 | 1.81 ± 0.99 | - | - | - | - |
| **[52]** | | 2.83 | 9.37 | 5.34 ± 1.44 | - | - | - | - | - | - |
| **[59]** | | 1.2 | 7.3 | 3.3 ± 1.2 | 3.4 ± 1.3 | 3.1 ± 1.1 | - | - | 3.4 ± 1.3 | 3.1 ± 1.1 |
| **[55]** | | 0.52 | 3.92 | - | - | - | - | - | - | - |

^†^: Values calculated based on data from publications
